# Differential Effects of 25-Hydroxyvitamin D_3_ versus 1α 25-Dihydroxyvitamin D_3_ on Adipose Tissue Browning in CKD-Associated Cachexia

**DOI:** 10.3390/cells10123382

**Published:** 2021-12-01

**Authors:** Robert H. Mak, Uwe Querfeld, Alex Gonzalez, Sujana Gunta, Wai W. Cheung

**Affiliations:** 1 Division of Pediatric Nephrology, Rady Children’s Hospital, University of California, San Diego, CA 92093, USA; alg022@health.ucsd.edu (A.G.); sujana.kaushik@gmail.com (S.G.); w5cheung@health.ucsd.edu (W.W.C.); 2 Department of Paediatric Gastroenterology, Nephrology and Metabolic Diseases, Charité-Universitätsmedizin Berlin, Augustenburger Platz 1, 13353 Berlin, Germany; uwe.querfeld@charite.de; 3 Pediatric Services, Vista Community Clinic, Vista, CA 92084, USA

**Keywords:** chronic kidney disease, vitamin D insufficiency, 25-hydroxyvitamin D_3_, 1α, 25-dihydroxyvitamin D_3_, cachexia, adipose tissue browning, muscle wasting

## Abstract

Patients with chronic kidney disease (CKD) often have low serum concentrations of 25(OH)D_3_ and 1,25(OH)_2_D_3_. We investigated the differential effects of 25(OH)D_3_ versus 1,25(OH)_2_D_3_ repletion in mice with surgically induced CKD. Intraperitoneal supplementation of 25(OH)D_3_ (75 μg/kg/day) or 1,25(OH)_2_D_3_ (60 ng/kg/day) for 6 weeks normalized serum 25(OH)D_3_ or 1,25(OH)_2_D_3_ concentrations in CKD mice, respectively. Repletion of 25(OH)D_3_ normalized appetite, significantly improved weight gain, increased fat and lean mass content and in vivo muscle function, as well as attenuated elevated resting metabolic rate relative to repletion of 1,25(OH)_2_D_3_ in CKD mice. Repletion of 25(OH)D_3_ in CKD mice attenuated adipose tissue browning as well as ameliorated perturbations of energy homeostasis in adipose tissue and skeletal muscle, whereas repletion of 1,25(OH)_2_D_3_ did not. Significant improvement of muscle fiber size and normalization of fat infiltration of gastrocnemius was apparent with repletion of 25(OH)D_3_ but not with 1,25(OH)_2_D_3_ in CKD mice. This was accompanied by attenuation of the aberrant gene expression of muscle mass regulatory signaling, molecular pathways related to muscle fibrosis as well as muscle expression profile associated with skeletal muscle wasting in CKD mice. Our findings provide evidence that repletion of 25(OH)D_3_ exerts metabolic advantages over repletion of 1,25(OH)_2_D_3_ by attenuating adipose tissue browning and muscle wasting in CKD mice.

## 1. Introduction

Cachexia in chronic kidney disease (CKD) is a complex metabolic disorder that results in profound loss of adipose tissue and muscle mass [1,2]. Adipose tissue is a critical metabolic and endocrine organ that regulates whole-body energy metabolism. The white adipose tissue (WAT) is a key energy reservoir while brown adipose tissue (BAT) is involved in the regulation of thermogenesis [3,4]. Recent studies have demonstrated that WAT browning, a process characterized by a phenotypic transition from WAT to thermogenic BAT, is implicated in the pathogenesis of cachexia. Indeed, browning of WAT preceded skeletal muscle atrophy in several disease models [5,6,7,8]. These results reveal the detrimental effects of WAT browning in the setting of disease-associated cachexia. Therefore, inhibition of WAT browning may exhibit therapeutic potential for patients with CKD-associated cachexia. Patients with CKD often exhibit low serum concentrations of 25(OH)D_3_ and 1,25(OH)_2_D_3_ [9,10,11]. We reported that combined supplementation of 25(OH)D_3_ and 1,25(OH)_2_D_3_ attenuated WAT browning and cachexia in CKD mice [12]. 25(OH)D_3_ is the most prevalent vitamin D metabolite and is often considered as biologically inactive until renal 1α-hydroxylation to the active hormone 1,25(OH)_2_D_3,_ which exerts its actions via the nuclear vitamin D receptor (VDR) [9,10,11]. However, recent studies show that 25(OH)D_3_ is also biologically active and exhibits potent anabolic effects in vivo and ex vivo [13,14,15,16,17,18]. In this study, we assess the differential effects of 25(OH)D_3_ versus 1,25(OH)_2_D_3_ repletion in CKD mice, with emphasis on WAT browning and muscle wasting.

## 2. Materials and Methods

### 2.1. Study Design

**Figure 1:**
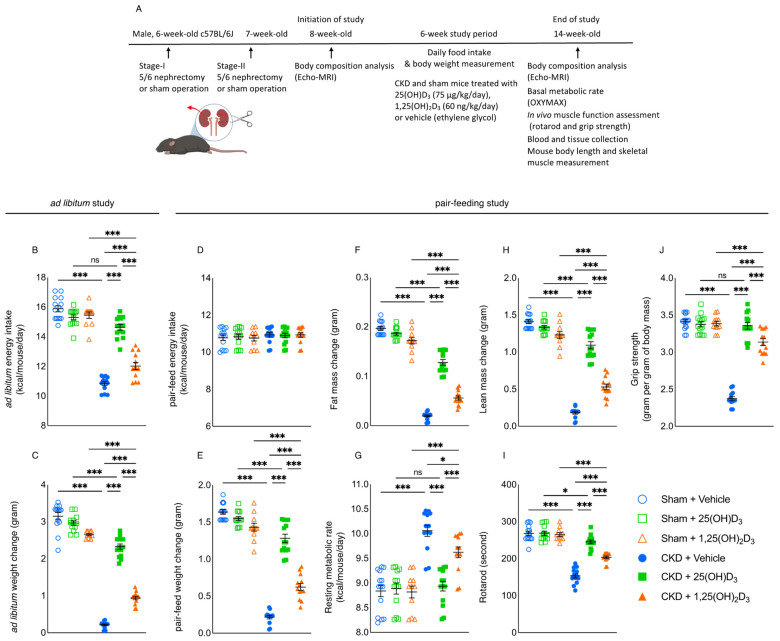
Repletion of 25(OH)D_3_ attenuates cachexia in CKD mice. We have performed two studies. 1. CKD and sham mice were given 25(OH)D_3_ (75 µg/kg/day), 1,25(OH)_2_D_3_ (60 ng/kg/day) or vehicle (ethylene glycol), respectively, for 6 weeks (**A**). All mice were fed *ad libitum*. We calculated *ad libitum* caloric intake (**B**) and recorded weight change in mice (**C**). 2. In another experiment, to assess the differential effects of 25(OH)D_3_ versus 1,25(OH)_2_D_3_ repletion beyond its nutritional effects, we employed a pair-feeding strategy. CKD + Vehicle mice were given an *ad libitum* amount of food whereas other groups of mice were given an equivalent amount of food (**D**). Weight gain, fat and lean content, resting metabolic rate, and in vivo muscle function (rotarod and grip strength) were measured in mice (**E**–**J**). Data are expressed as mean ± SEM. Results of CKD + Vehicle, CKD + 25(OH)D_3_ and CKD + 1,25(OH)_2_D_3_ mice were compared to those of Sham + Vehicle, Sham + 25(OH)D_3_ and Sham + 1,25(OH)_2_D_3_ mice, respectively. In addition, results of CKD + Vehicle were compared to those of CKD + 25(OH)D_3_ and CKD + 1,25(OH)_2_D_3_ mice, respectively. Furthermore, results of CKD + 25(OH)D_3_ mice were compared to those of CKD + 1,25(OH)_2_D_3_ mice. Specific *p*-values are shown above the bar. ns: signifies not significant, ^∗^ *p* < 0.05, ^∗∗∗^ *p* < 0.001.

This study was conducted in compliance with established guidelines and prevailing protocol (S01754) as approved by the Institutional Animal Care and Use Committee (IACUC) at the University of California, San Diego in accordance with the National Institutes of Health, Bethesda, MD, USA. Male c57BL/6 J mice at 6 weeks of age were used for the study. CKD in mice was surgically induced by two-stage 5/6 nephrectomy while a sham procedure was performed in control mice [12]. Mice were housed with 12:12 h light-dark cycles with *ad libitum* access to mouse diet 5015 (LabDiet, St Louis, MO, USA, catalog 0001328, with a metabolizable energy value of 3.59 kcal/g) and water prior to the initiation of the experiment. We have performed a series of experiments to determine the minimum dose of 25(OH)D_3_, and 1,25(OH)_2_D_3_ in order to replenish the serum levels of those molecules in CKD mice. CKD mice were treated with 25(OH)D_3_ (Sigma, Northbrook, IL, USA, Catalog 739,650-1ML, 25, 50 or 75 μg/kg/day), 1,25(OH)_2_D_3_ (Sigma, Northbrook, IL, USA, Catalog 740,578-1ML, 20, 40 or 60 ng/kg/day) or vehicle (ethylene glycol) using a subcutaneous osmotic Alzet mini-osmotic pump model 2006 (Durect Corporation, Cupertino, CA, USA). Results are shown in Table 1, Table 2 and Table 3. Subsequently, we have performed the following two studies to investigate the differential effects of 25(OH)D_3_ versus 1,25(OH)_2_D_3_ repletion in CKD mice. The schematic study plan for these two studies is illustrated in Figure 1A. Study 1—CKD and sham mice were treated with 25(OH)D_3_ (75 μg/kg/day), 1,25(OH)_2_D_3_ (60 ng/kg/day), or vehicle. The study period was 6 weeks and all mice were housed in individual cages and had free access to the rodent diet. Mice were sacrificed at 14 weeks of age. We compared caloric intake and weight change in CKD and sham mice. The caloric intake for each mouse was calculated by multiplying total mouse diet consumption (in grams) with the metabolizable energy value of the diet (3.59 kcal/g). The result for energy intake is expressed as kcal/mouse/day. Study 2—we evaluated the effects of 25(OH)D_3_ and 1,25(OH)_2_D_3_ in CKD mice, beyond nutritional stimulation by employing a pair-feeding strategy. CKD and sham mice were given 25(OH)D_3_ (75 μg/kg/day), 1,25(OH)_2_D_3_ (60 ng/kg/day) or vehicle for 6 weeks. Vehicle-treated CKD mice were fed *ad libitum* while all other groups of mice were fed an equal amount of rodent diet based on the recorded food intake of vehicle-treated CKD mice. Each mouse was housed in an individual cage. We measured the weight change for each mouse. Mice were sacrificed at 14-weeks of age.

### 2.2. Body Composition Analysis

Body composition (for lean and fat content) was measured by quantitative magnetic resonance analysis (EchoMRI-100^TM^, Echo Medical System, Hoston, TX, USA) [12]. All measurements were made during the light phase (0900–1900). Procedures were performed according to the manufacturer’s instructions. Contents of whole-body fat, lean mass, free water, and total body water were calculated.

### 2.3. Resting Metabolic Rate

Indirect calorimetry was performed in mice using Oxymax calorimetry (Columbus Instruments, Columbus, OH, USA) during the daytime (9 a.m. to 5 p.m.). Oxygen (VO_2_) and carbon dioxide (VCO_2_) consumption were simultaneously measured. The respiratory exchange ratio (RER) was calculated as the quotient VCO_2_/VO_2_. Energy expenditure was measured as the production of kilocalories of heat and was calculated as Caloric Value (CV) × VO_2_ where CV is 3.815 + 1.232 × RER. Resting metabolic rate during the daytime (9 a.m. to 5 p.m.) in an individual mouse is expressed as kcal/mouse/day [19].

### 2.4. Mouse Muscle Function

Measurements of muscle function were recorded at the end of the study. All measurements were conducted by one trained investigator. Rotarod performance was utilized to assess neuromuscular coordination at the end of the study. All mice underwent three days of acclimatization prior to data collection. Mice were placed on the rod in the identical forward direction, and then the rotarod performance tool (model RRF/SP, Accuscan Instrument, Columbus, OH, USA) was started at 0 rpm and increased to 40 rpm at 0.4 rpm/s, after a 60 s acclimatization period at 4 rpm. The latency to fall from the rod was recorded in seconds with a maximum time of 300 s. This procedure was repeated for a total of 6 trials (three trials per day with >2 h of rest time between trials) over three days, and the average of the trials was used in the data analysis. Rotarod performance was reported as latency to fall in seconds [12,19]. For forelimb strength, holding the mice by the tail, the front feet were allowed to grip a grate, and then they were pulled from the grate, generating a force measured by the force transducer (Model 47106, UGO Basile, Gemonio, VA, Italy). Five measurements were taken, three days consecutively, with the first day used as acclimatization and not included in the final data analysis. The average of the measurements was used in the data analysis [12,19]. Grip strength was reported in grams of strength per gram of body mass. 

### 2.5. Serum and Blood Chemistry

The serum concentration of bicarbonate, Ca and Pi was assessed. Concentrations of BUN, 25(OH)D_3_, 1,25(OH)_2_D_3_, parathyroid hormone (PTH) and vitamin D binding protein (VDBP) were analyzed (Supplemental Table S1). Serum creatinine was analyzed by the LC-MS/MS method [20].

### 2.6. Protein Assay for Muscle and Adipose Tissue 

A portion of the right gastrocnemius muscle of mice, inguinal WAT and intercapsular BAT, were processed in a homogenizer tube (USA Scientific, Orlando, FL, USA, catalog 1420-9600) containing ceramic beads (Omni International, Kennesaw, GA, USA, catalog 19-646) using a Bead Mill Homogenizer (Omni International, Kennesaw, GA, USA). Protein concentration of tissue homogenate was assayed using a Pierce BAC Protein Assay Kit (Thermo Scientific, Waltham, MA, USA, catalog 23227). Uncoupling (UCP) protein content along with adenosine triphosphate (ATP) concentration in adipose tissue and muscle homogenates were assayed (Supplemental Table S1).

### 2.7. Fiber Size and Fatty Infiltration of Gastrocnemoius 

We processed the dissected left gastrocnemius according to an established protocol and measured muscle fiber cross-sectional area, using ImageJ software (https://rsbweb.nih.gob/ij/, accessed on 23 August 2021) [12,19]. We also quantified fatty infiltration in skeletal muscle. A portion of dissected left gastrocnemius was incubated with Oil Red O (Oil Red O Solution, catalog number O1391-250 mL, Sigma Aldrich, St. Louis, MO, USA) [21]. Detailed procedures for Oil Red O staining were in accordance with published protocol. Acquisition and quantification of images were analyzed using ImageJ software (https://rsbweb.nih.gob/ij/, accessed on 23 August 2021) [22].

### 2.8. RT^2^ Profiler PCR Array for Muscle Fibrosis

We characterized gastrocnemius muscle expression of 84 key genes involved in tissue fibrosis in 14-week-old CKD versus age-matched control mice, using RT^2^ Profiler PCR array (Qiagen, Germantown, MA, USA, Catalog 330,231 PAMM-120ZA). Detailed information for muscle mRNA extraction and subsequent reverse transcription and qPCR analysis of 84 genes were published [12]. We identified a total of 12 fibrotic genes that have been implicated in CKD-associated muscle fibrosis. In this study, we performed qPCR analysis for those 12 muscle fibrotic genes in the different experimental groups.

### 2.9. Muscle RNAseq Analysis

We performed RNAseq analysis on gastrocnemius muscle mRNA in 12-month-old CKD mice versus age-appropriate sham mice. Detailed procedures for mRNA extraction, purification and subsequent construction of cDNA libraries as well as analysis of gene expression were published [12]. We then performed Ingenuity Pathway Analysis enrichment tests for those differentially expressed muscle genes in 12-month-old CKD mice versus sham mice, focusing on pathways related to energy metabolism, skeletal and muscle system development and function, and organismal injury and abnormalities. We identified the top 12 differentially expressed muscle genes in 12-month-old CKD mice versus sham mice. In this study, we evaluated the differential effects of 25(OH)D_3_ versus 1,25(OH)_2_D_3_ repletion on the expression of these 12 differentially expressed gastrocnemius muscle genes for the following six groups of younger mice (3 months of age at sacrifice), i.e., Sham + Vehicle, Sham + 25(OH)D_3_, Sham + 1,25(OH)_2_D_3,_ CKD + Vehicle, CKD + 25(OH)D_3_ as well as CKD + 1,25(OH)_2_D_3_ by qPCR technique.

### 2.10. Quantative Real-Time PCR

A portion of the right gastrocnemius muscle of mice, inguinal WAT and intercapsular BAT was processed in a homogenizer tube (USA Scientific, Orlando, FL, USA, catalog 1420-9600) containing ceramic beads (Omni International, Kennesaw, GA, USA, catalog 19-646) using a Bead Mill Homogenizer (Omni International, Kennesaw, GA, USA). Total RNA from the gastrocnemius and adipose tissues was isolated using TriZol (Life Technology, Carslbad, CA, USA). Total RNA (3 µg) was reverse transcribed to cDNA with SuperScript III Reverse Transcriptase (Invitrogen, Waltham, MA, USA). Quantitative real-time RT-PCR of target genes was performed using KAPA SYBR FAST qPCR kit (KAPA Biosystems, Wilmington, MA, USA) [12,19]. Glyceraldehyde−3-phosphate dehydrogenase (GAPDH) was used as an internal control. Expression levels were calculated according to the relative 2^−ΔΔCt^ method. All primers are listed (Supplemental Table S2).

### 2.11. Statistics

Statistical analyses were performed using GraphPad Prism version 9.2.0. All data are presented as mean ± S.E.M. For comparison of the means between two groups, data were analyzed by Student’s 2-tailed *t*-test. Differences of the means for more than two groups containing two variables were analyzed using 2-way ANOVA. Post-hoc analysis was performed with Tukey’s test. A *p*-value less than 0.05 was considered significant.

## 3. Results

### 3.1. Supplementation of Vitamin D Replenishes Serum Vitamin D Levels in CKD Mice 

We carried out a series of experiments to determine the minimum dose of 25(OH)D_3_, and 1,25(OH)_2_D_3_ in order to replenish the serum level of those molecules in CKD mice. CKD in mice was induced by a two-stage sub-total nephrectomy. CKD and sham mice were initially treated with 25(OH)D_3_ (25 or 50 µg/kg per day), 1,25(OH)_2_D_3_ (20 or 40 ng/kg per day), or ethylene glycol as a vehicle for 6 weeks. Serum and blood chemistry of CKD mice after 6 weeks of vitamin D supplementation were listed (Table 1 and Table 2, respectively). The serum concentration of 25(OH)D_3_ and 1,25(OH)_2_D_3_ were increased in CKD + 25(OH)D_3_ and CKD + 1,25(OH)_2_D_3_ relative to CKD + Vehicle mice, respectively, but still lower than that in Sham + Vehicle mice. Importantly, CKD + 25(OH)D_3_ (75 µg/kg per day) and CKD + 1,25(OH)_2_D_3_ (60 ng/kg per day) had comparable serum 25(OH)D_3_ and 1,25(OH)_2_D_3_ concentrations as those in Sham + Vehicle mice (Table 3).

### 3.2. Repletion of 25-Hydroxyvitamin D_3_ Normalizes Caloric Intake and Improves Weight Gain in CKD Mice 

We then performed the following two experiments to investigate the differential effects of 25(OH)D_3_ versus 1,25(OH)_2_D_3_ on adipose tissue browning in CKD-associated cachexia. For the first study, we showed that supplementation of 25(OH)D_3_ (75 µg/kg per day) or 1,25(OH)_2_D_3_ (60 ng/kg per day) respectively normalized serum 25(OH)D_3_ or 1,25(OH)_2_D_3_ concentrations in CKD mice (Table 4). Furthermore, supplementation of 25(OH)D_3_ significantly increased serum 1,25(OH)_2_D_3_ concentration in CKD mice. We studied the dietary effects of 25(OH)D_3_ or 1,25(OH)_2_D_3_ repletion in CKD mice. Mice were fed *ad libitum*. Repletion of 25(OH)D_3_ corrected anorexia in CKD mice while supplementation of 1,25(OH)_2_D_3_ did not (Figure 1B). Repletion of 25(OH)D_3_ significantly improved weight gain relative to repletion of 1,25(OH)_2_D_3_ in CKD mice (Figure 1C). 

### 3.3. Repletion of 25-Hydroxyvitamin D_3_ Improves Energy Homeostasis in CKD Mice

For the second study, we compared the differential effects of 25(OH)D_3_ versus 1,25(OH)_2_D_3_ repletion in CKD mice beyond appetite stimulation by employing a food restrictive strategy. CKD and sham mice were given 25(OH)D_3_ (75 µg/kg/day), 1,25(OH)_2_D_3_ (60 ng/kg/day) or vehicle control for 6 weeks. CKD + Vehicle mice were fed *ad libitum*. Daily *ad libitum* caloric intake for CKD + Vehicle mice was measured. Subsequently, all other groups of mice were given the equivalent amount of energy intake as those of CKD + Vehicle (Figure 1D). At the end of the study, mice were sacrificed, and we measured the serum and blood chemistry of mice. Importantly, supplementation of 25(OH)D_3_ repleted serum concentration of 25(OH)D_3_ as well as significantly increased serum 1,25(OH)_2_D_3_ concentration in CKD mice (Table 5). Increased serum PTH concentrations were observed in CKD mice versus sham mice. Repletion of 25(OH)D_3_ or 1,25(OH)_2_D_3_ caused a tendency of decreased serum concentrations of PTH in CKD mice although it did not reach statistical significance. In addition, serum concentrations of vitamin D binding protein (VDBP) were elevated in CKD mice versus sham mice. Repletion of 25(OH)D_3_ or 1,25(OH)_2_D_3_ did not normalize serum VDBP levels in CKD mice. Repletion of 25(OH)D_3_ normalized resting metabolic rate and grip strength in CKD mice while repletion of 1,25(OH)_2_D_3_ did not (Figure 1G,J). Moreover, repletion of 25(OH)D_3_ significantly increased weight gain, fat mass and lean mass content as well as improved rotarod activity than repletion of 1,25(OH)_2_D_3_ in CKD mice (Figure 1E,F,H,I).

### 3.4. Repletion of 25-Hydroxyvitamin D_3_ Attenuates Adipose Tissue and Skeletal Muscle Energy Homeostasis in CKD Mice

**Figure 2:**
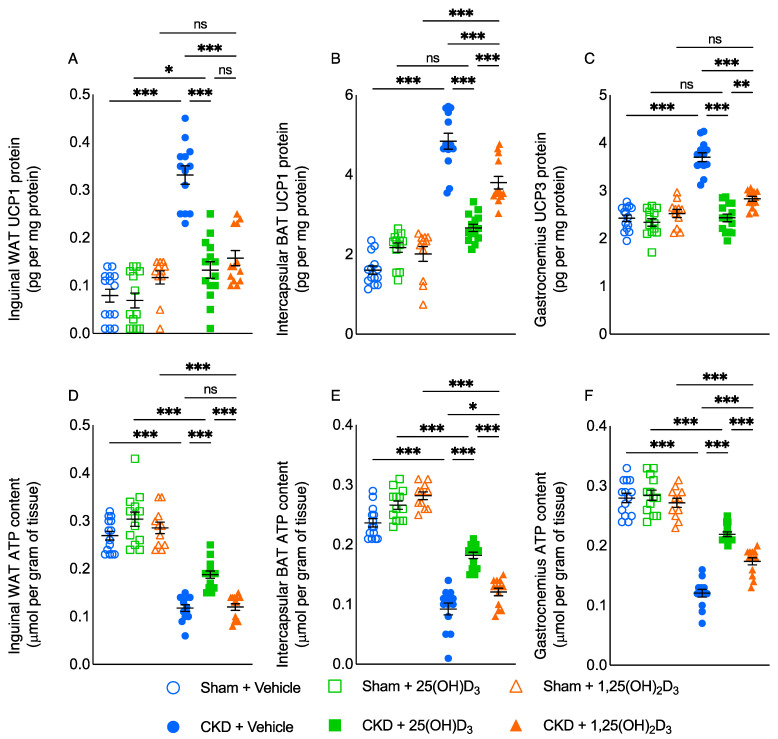
Repletion of 25-hydroxyvitamin D_3_ ameliorates energy homeostasis in adipose tissue and skeletal muscle in CKD mice. UCP and ATP content in adipose tissue (inguinal WAT and intercapsular BAT) and gastrocnemius muscle was measured. Data are expressed as mean ± SEM. Results of CKD + Vehicle, CKD + 25(OH)D_3_ and CKD + 1,25(OH)_2_D_3_ mice were compared to those of Sham + Vehicle, Sham + 25(OH)D_3_ and Sham + 1,25(OH)_2_D_3_ mice, respectively. In addition, results of CKD + Vehicle were compared to those of CKD + 25(OH)D_3_ and CKD + 1,25(OH)_2_D_3_ mice, respectively. Furthermore, results of CKD + 25(OH)D_3_ mice were compared to those of CKD + 1,25(OH)_2_D_3_ mice. Specific *p*-values are shown above the bar. ns: signifies not significant, ^∗^ *p* < 0.05, ^∗∗^ *p* < 0.01, ^∗∗∗^ *p* < 0.001.

Metabolism of adipose tissue and skeletal muscle influences basal metabolic rate and energy expenditure, which can substantially affect whole-body metabolism and weight gain [23]. We studied the effects of vitamin D repletion on adipose tissue and skeletal muscle energy homeostasis in CKD mice. The protein content of UCPs in WAT, BAT as well as gastrocnemius was significantly higher in CKD mice (Figure 2). In contrast, ATP content in WAT, BAT and gastrocnemius was significantly lower in CKD mice. Repletion of 25(OH)D_3_ normalized UCP1 and gastrocnemius UCP3 content in CKD mice while repletion of 1,25(OH)_2_D_3_ did not (Figure 2A–C). In addition, repletion of 25(OH)D_3_ significantly improved ATP content in WAT, BAT, and gastrocnemius relative to repletion of 1,25(OH)_2_D_3_ in CKD mice (Figure 2D–F). 

### 3.5. Repletion of 25-Hydroxyvitamin D_3_ Attenuates Browning of White Adipose Tissue in CKD Mice

**Figure 3:**
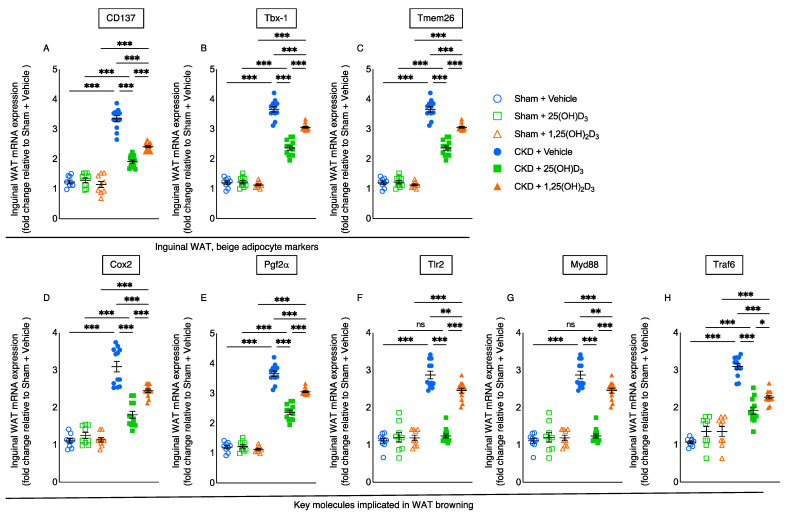
Repletion of 25-hydroxyvitamin D_3_ attenuates white adipose tissue browning in CKD mice. Gene expression of beige adipocyte markers (CD137, Tbx−1 and Tmem26) and important molecules mediate WAT browning (Cox2, Pgf2α, Tlr2, Myd88 and Traf6) in inguinal WAT was measured by qPCR. Final results were expressed in arbitrary units, with one unit being the mean level in Sham + Vehicle mice. Data are expressed as mean ± SEM. Results of CKD + Vehicle, CKD + 25(OH)D_3_ and CKD + 1,25(OH)_2_D_3_ mice were compared to those of Sham + Vehicle, Sham + 25(OH)D_3_ and Sham + 1,25(OH)_2_D_3_ mice, respectively. In addition, results of CKD + Vehicle were compared to those of CKD + 25(OH)D_3_ and CKD + 1,25(OH)_2_D_3_ mice, respectively. Furthermore, results of CKD + 25(OH)D_3_ mice were compared to those of CKD + 1,25(OH)_2_D_3_ mice. Specific *p*-values are shown above the bar. ns: signifies not significant, ^∗^ *p* < 0.05, ^∗∗^ *p* < 0.01, ^∗∗∗^ *p* < 0.001.

Recent data suggest that WAT browning contributes to energy-wasting in cachexia. White, beige and brown adipocytes are distinct but often occur mixed together within individual depots [3,4,5]. In CKD mice, repletion of 25(OH)D_3_ significantly decreased inguinal WAT expression of beige adipocyte cell surface markers (CD137, Tbx1 and Tmem26), and the effect was significantly stronger than observed with 1,25(OH)_2_D_3_ repletion (Figure 3A–C). Cox2/Pgf2α induces *de novo* browning recruitment in WAT. Activation of toll-like receptor Tlr2 and their adaptor molecules, such as Myd88 and Traf6 stimulate browning of WAT [24]. We showed that repletion of 25(OH)D_3_ significantly decreased inguinal WAT expression of Cox2, Pgf2α, Tlr2, Myd88 and Traf6 in CKD mice and that the effect was significantly stronger than seen with 1,25(OH)_2_D_3_ repletion in CKD mice (Figure 3D–H).

### 3.6. Repletion of 25-Hydroxyvitamin D_3_ Attenuates WAT Thermogenic Gene Expression in CKD Mice

**Figure 4:**
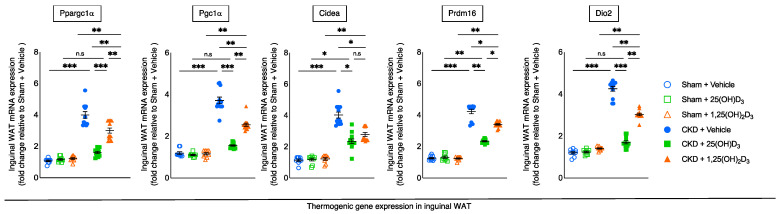
Repletion of 25-hydroxyvitamin D_3_ attenuates white adipose tissue thermogenic gene expression in CKD mice. Gene expression of thermogenic gene (Ppargc1α, Pgc1α, Cidea, Prdm16 and Dio2) in inguinal WAT was measured by qPCR. Final results were expressed in arbitrary units, with one unit being the mean level in Sham + Vehicle mice. Data are expressed as mean ± SEM. Results of CKD + Vehicle, CKD + 25(OH)D_3_ and CKD + 1,25(OH)_2_D_3_ mice were compared to those of Sham + Vehicle, Sham + 25(OH)D_3_ and Sham + 1,25(OH)_2_D_3_ mice, respectively. In addition, results of CKD + Vehicle were compared to those of CKD + 25(OH)D_3_ and CKD + 1,25(OH)_2_D_3_ mice, respectively. Furthermore, results of CKD + 25(OH)D_3_ mice were compared to those of CKD + 1,25(OH)_2_D_3_ mice. Specific *p*-values are shown above the bar. ns: signifies not significant, ^∗^ *p* < 0.05, ^∗∗^ *p* < 0.01, ^∗∗∗^ *p* < 0.001.

Inguinal WAT of CKD mice displayed significantly increased thermogenesis gene expression (Ppargc1α, Pgc1α, Cidea, Prdm16, and Dio2) relative to sham mice. Repletion of 25(OH)D_3_ normalized inguinal WAT (Ppargc1α, Pgc1α, and Dio2) or attenuated (Cidea and Prdm16) gene expression in CKD mice (Figure 4).

### 3.7. Repletion of 25-Hydroxyvitamin D_3_ Attenuates Muscle Wasting Signaling Pathways in CKD Mice

**Figure 5:**
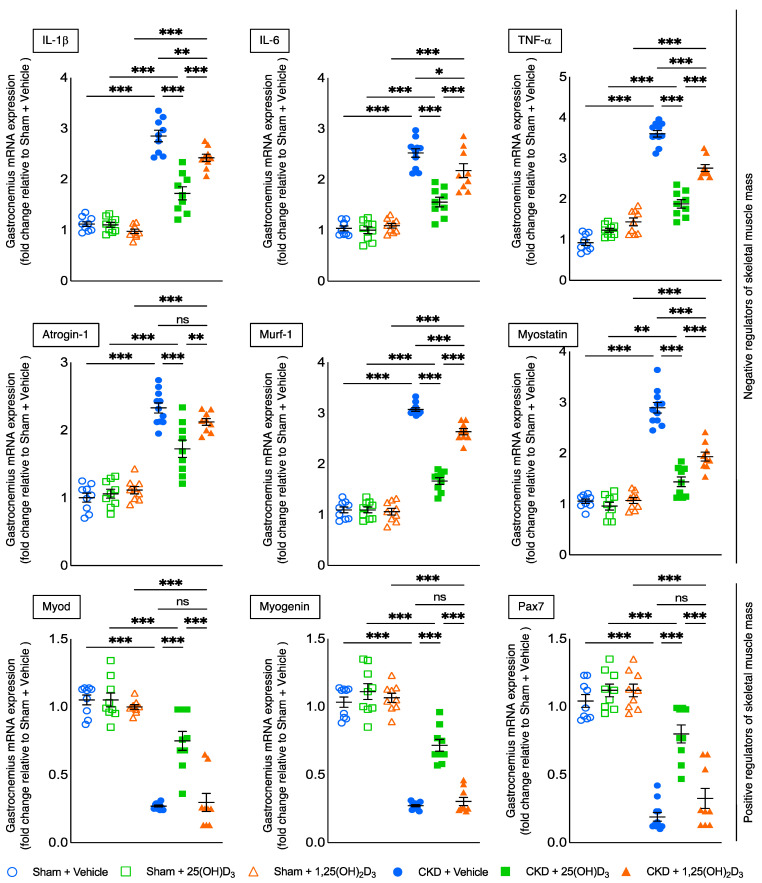
Repletion of 25-hydroxyvitamin D_3_ attenuates signaling pathways implicated in muscle wasting in CKD mice. Gastrocnemius muscle expression of negative regulators of skeletal muscle mass (Atrogin-1, Murf-1, Myostatin, IL-1β, IL-6 and TNF-α), as well as pro-myogenic factors (Myod, Myogenin and Pax7), were measured by qPCR. Final results were expressed in arbitrary units, with one unit being the mean level in Sham + Vehicle mice. Data are expressed as mean ± SEM. Results of CKD + Vehicle, CKD + 25(OH)D_3_ and CKD + 1,25(OH)_2_D_3_ mice were compared to those of Sham + Vehicle, Sham + 25(OH)D_3_ and Sham + 1,25(OH)_2_D_3_ mice, respectively. In addition, results of CKD + Vehicle were compared to those of CKD + 25(OH)D_3_ and CKD + 1,25(OH)_2_D_3_ mice, respectively. Furthermore, results of CKD + 25(OH)D_3_ mice were compared to those of CKD + 1,25(OH)_2_D_3_ mice. Specific *p*-values are shown above the bar. ns: signifies not significant, ^∗^ *p* < 0.05, ^∗∗^ *p* < 0.01, ^∗∗∗^ *p* < 0.001.

Inflammatory cytokines induce muscle atrophy [1,2]. Repletion of 25(OH)D_3_ attenuated gastrocnemius expression of inflammatory cytokines (IL-1β, IL-6 and TNF-α) in CKD mice. In addition, repletion of 25(OH)D_3_ in CKD mice significantly decreased gastrocnemius expression of negative regulators of skeletal muscle mass (Atrogin-1, Murf-1, Myostatin), and the effect was significantly stronger than seen with 1,25(OH)_2_D_3_ repletion. Furthermore, repletion of 25(OH)D_3_ increased the expression of the positive regulators of skeletal muscle mass (Myod, Myogenin and Pax-7) whereas repletion of 1,25(OH)_2_D_3_ had no significant effect (Figure 5).

### 3.8. Repletion of 25-Hydroxyvitamin D_3_ Increases Muscle Fiber Size in CKD Mice

**Figure 6:**
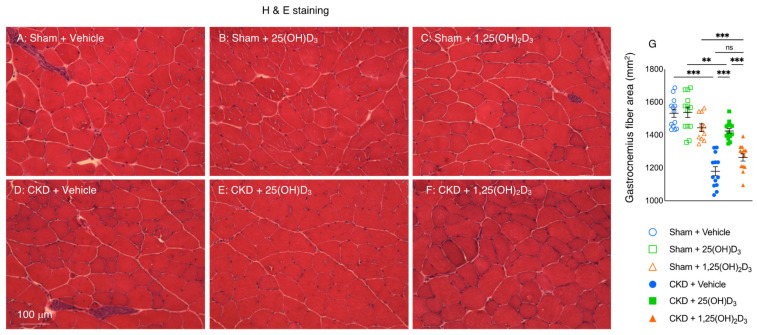
Repletion of 25-hydroxyvitamin D_3_ increases gastrocnemius fiber size in CKD mice. Representative photomicrographs of the gastrocnemius with H&E staining (**A**–**F**). Average gastrocnemius cross-sectional area was measured (**G**). Data are expressed as mean ± SEM. Results of CKD + Vehicle, CKD + 25(OH)D_3_ and CKD + 1,25(OH)_2_D_3_ mice were compared to those of Sham + Vehicle, Sham + 25(OH)D_3_ and Sham + 1,25(OH)_2_D_3_ mice, respectively. In addition, results of CKD + Vehicle were compared to those of CKD + 25(OH)D_3_ and CKD + 1,25(OH)_2_D_3_ mice, respectively. Furthermore, results of CKD + 25(OH)D_3_ mice were compared to those of CKD + 1,25(OH)_2_D_3_ mice. Specific *p*-values are shown above the bar. ns: signifies not significant, ^∗∗^ *p* < 0.01, ^∗∗∗^ *p* < 0.001.

We studied the effect of vitamin D repletion on skeletal muscle morphology in CKD mice. Repletion of 25(OH)D_3_ significantly increased the average cross-sectional area of the gastrocnemius in CKD mice while repletion of 1,25(OH)_2_D_3_ did not (Figure 6).

### 3.9. Repletion of 25-Hydroxyvitamin D_3_ Normalizes Muscle Fat Infiltration in CKD Mice

**Figure 7:**
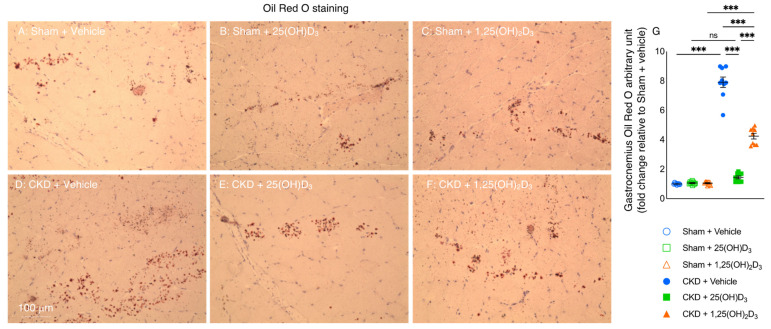
Repletion of 25-hydroxyvitamin D_3_ normalizes muscle fat infiltration in CKD mice. Visualization of the quantification of fatty infiltration by Oil Red O analysis in the gastrocnemius muscle (**A**–**F**). Final results were expressed in arbitrary units, with one unit being the mean staining intensity in Sham + Vehicle mice (**G**). Data are expressed as mean ± SEM. Results of CKD + Vehicle, CKD + 25(OH)D_3_ and CKD + 1,25(OH)_2_D_3_ mice were compared to those of Sham + Vehicle, Sham + 25(OH)D_3_ and Sham + 1,25(OH)_2_D_3_ mice, respectively. In addition, results of CKD + Vehicle were compared to those of CKD + 25(OH)D_3_ and CKD + 1,25(OH)_2_D_3_ mice, respectively. Furthermore, results of CKD + 25(OH)D_3_ mice were compared to those of CKD + 1,25(OH)_2_D_3_ mice. Specific *p*-values are shown above the bar. ns: signifies not significant, ^∗∗∗^ *p* < 0.001.

Fatty infiltration of the skeletal muscle is a common and important feature of myopathies. We showed that repletion of 25(OH)D_3_ normalized fatty infiltration in skeletal muscle in CKD mice while repletion of 1,25(OH)_2_D_3_ did not (Figure 7).

### 3.10. Repletion of 25-Hydroxyvitamin D_3_ Attenuates Aberrant Muscle Fibrotic Gene Expression in CKD Mice

**Figure 8:**
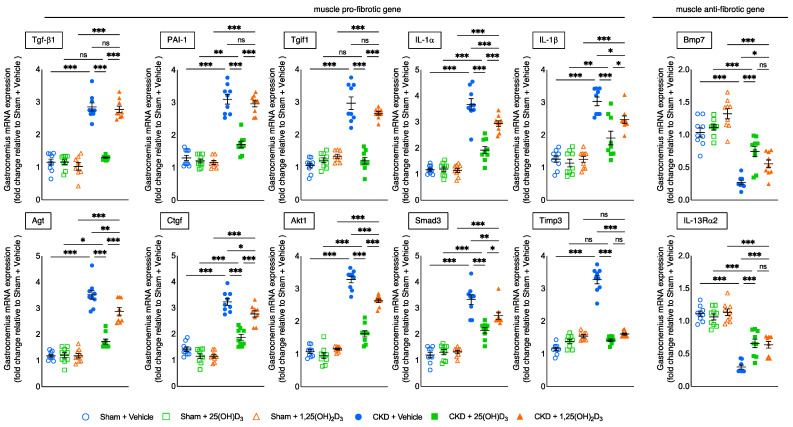
Repletion of 25-hydroxyvitamin D_3_ attenuates gastrocnemius muscle fibrotic gene expression in CKD mice. Repletion of 25-hydroxyvitamin D_3_ normalized muscle expression of pro-fibrotic genes (Tgfα1, Tgif1, Timp3) as well as attenuated pro-fibrotic genes (PAI-1, IL-1α, IL-1β, Agt, Ctgf, Akt1 and Smad3) and anti-fibrotic genes (Bmp7 and IL-13Rα2) of the gastrocnemius in CKD + 25(OH)D_3_ mice. Gene expression of fibrotic genes in gastrocnemius muscle was measured by qPCR. Final results were expressed in arbitrary units, with one unit being the mean level in Sham + Vehicle mice. Data are expressed as mean ± SEM. Results of CKD + Vehicle, CKD + 25(OH)D_3_ and CKD + 1,25(OH)_2_D_3_ mice were compared to those of Sham + Vehicle, Sham + 25(OH)D_3_ and Sham + 1,25(OH)_2_D_3_ mice, respectively. In addition, results of CKD + Vehicle were compared to those of CKD + 25(OH)D_3_ and CKD + 1,25(OH)_2_D_3_ mice, respectively. Furthermore, results of CKD + 25(OH)D_3_ mice were compared to those of CKD + 1,25(OH)_2_D_3_ mice. Specific *p*-values are shown above the bar. ns: signifies not significant, ^∗^ *p* < 0.05, ^∗∗^ *p* < 0.01, ^∗∗∗^ *p* < 0.001.

We profiled gastrocnemius muscle expression of 84 key genes involved in tissue fibrosis in CKD mice. We found that 10 pro-fibrotic genes (Tgfα1, PAI-1, Tgif1, IL-1α, IL-1β, Agt, Ctgf, Akt1, Smad3 and Timp3) were upregulated while two anti-fibrotic genes (Bmp7 and IL-13Rα2) were downregulated in 14-week-old CKD mice versus sham mice [12]. In this study, we performed qPCR analysis for those 12 muscle fibrotic genes in the different experimental groups. Repletion of 25(OH)D_3_ normalized muscle expression of pro-fibrotic genes (Tgfα1 and Tgif1) in CKD mice while repletion of 1,25(OH)_2_D_3_ did not (Figure 8). Moreover, repletion of 25(OH)D_3_ in CKD mice significantly decreased expression of pro-fibrotic genes (PAI-1, IL-1α, IL-1β, Agt, Ctgf, Akt1 and Smad3) and increased the expression of anti-fibrotic genes (Bmp7 and IL-13Rα2) the repletion of 1,25(OH)_2_D_3_. 

### 3.11. Molecular Mechanism of 25-Hydroxyvitamin D_3_ Repletion by RNAseq Analysis

**Figure 9:**
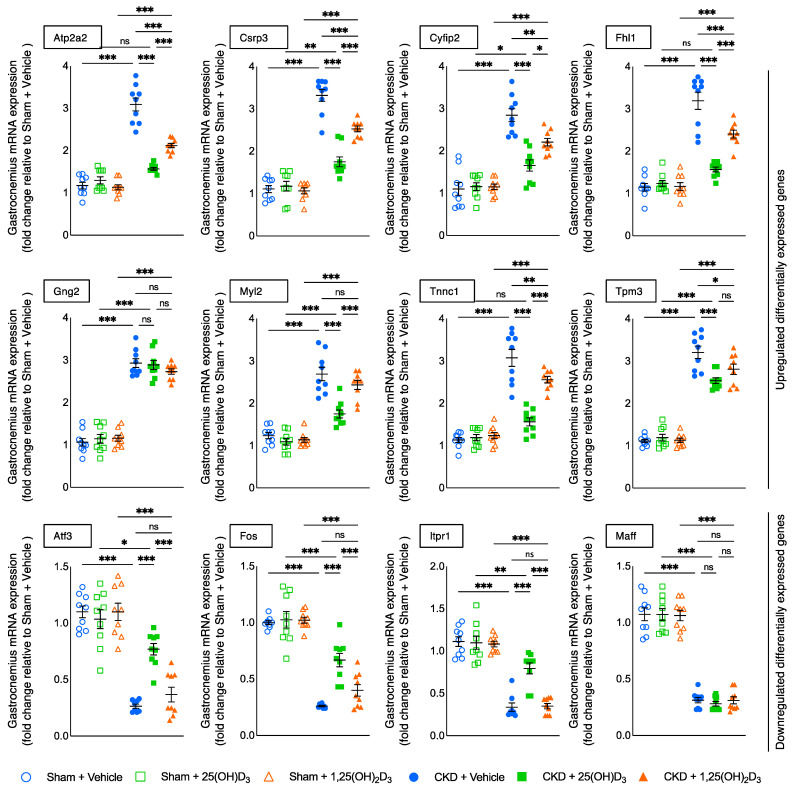
Repletion of 25-hydroxyvitamin D_3_ attenuates expression of gastrocnemius muscle genes in CKD mice. Repletion of 25-hydroxyvitamin D_3_ attenuated or normalized (Atp2a2, Csrp3, Cyfip2, Fhl1, Myl2, Tnnc1) as well as (Atf3, Fos and Itpr1) muscle gene expression in CKD + 25(OH)D_3_ mice. Nonsignificant changes were observed in Gng2, Tpm3, and Maff muscle gene expression in CKD + 25(OH)D_3_ mice relative to CKD + 1,25(OH)_2_D_3_ mice. Gene expression of target molecules in gastrocnemius muscle was measured by qPCR. Final results were expressed in arbitrary units, with one unit being the mean level in Sham + Vehicle mice. Data are expressed as mean ± SEM. Results of CKD + Vehicle, CKD + 25(OH)D_3_ and CKD + 1,25(OH)_2_D_3_ mice were compared to those of Sham + Vehicle, Sham + 25(OH)D_3_ and Sham + 1,25(OH)_2_D_3_ mice, respectively. In addition, results of CKD + Vehicle were compared to those of CKD + 25(OH)D_3_ and CKD + 1,25(OH)_2_D_3_ mice, respectively. Furthermore, results of CKD + 25(OH)D_3_ mice were compared to those of CKD + 1,25(OH)_2_D_3_ mice. Specific *p*-values are shown above the bar. ns: signifies not significant, ^∗^ *p* < 0.05, ^∗∗^ *p* < 0.01, ^∗∗∗^ *p* < 0.001.

Previously, we performed gastrocnemius RNAseq analysis between 12-month-old CKD and control mice and identified the top twelve differentially expressed genes that have been associated with energy metabolism, skeletal and muscular system development, and function [12]. Upregulated genes included Atp2a2, Csrp3, Cyfip2, Fhl1, Gng2, Myl2, Tnnc1 and Tpm3 whereas downregulated genes were Atf3, Fos, Itpr1 and Maff in 12-month-old CKD mice versus age-appropriate sham mice. In this study we examined the effects of vitamin D repletion in the different experimental groups (three months of age at sacrifice), focusing on gastrocnemius expression of these top 12 differentially expressed genes previously identified. Repletion of 25(OH)D_3_ normalized (Atp2a2, Fhl1, Tnnc1) expression in CKD mice (Figure 9). In addition, the repletion of 25(OH)D_3_ significantly decreased the expression of upregulated genes (Csrp3, Cyfip2, Myl2) and increased the expression of downregulated genes (Atf3, Fos, Itpr1) relative to repletion of 1,25(OH)_2_D_3_ in CKD mice. Nonsignificant changes were observed in Gng2, Tpm3, and Maff gene expression. The functional significance of these differentially expressed muscle genes were summarized in Table 6.

## 4. Discussion

Vitamin D has a long-established role in bone health. Recently, there has been evidence for the non-skeletal beneficial effects of vitamin D. In this study, we investigated the differential effects 25(OH)D_3_ versus 1,25(OH)_2_D_3_ repletion on fat and muscle in a mouse model of CKD cachexia. The novelty of this study is that repletion of 25(OH)D_3_ exhibited important metabolic advantages over repletion of 1,25(OH)_2_D_3_. Intraperitoneal supplementation of 25(OH)D_3_ and 1,25(OH)_2_D_3_ normalized serum concentrations of 25(OH)D_3_ and 1,25(OH)_2_D_3,_ respectively, in CKD mice. Importantly, we showed that repletion of 25(OH)D_3_ corrected anorexia and attenuated WAT browning and muscle wasting in CKD mice.

Metabolic advantages of repletion 25(OH)D_3_ over 1,25(OH)_2_D_3_ are likely due to the following mechanisms. Intracrine and localized, tissue-specific, conversion of 25(OH)D_3_ to 1,25(OH)_2_D_3_ is likely the major driving force behind 25(OH)D_3_ action, resulting in positive non-skeletal effects on human health [39]. Moreover, cellular uptake of 25(OH)D_3_ is potentially greater than that of 1,25(OH)_2_D_3_ due to the higher hydrophobicity of 25(OH)D_3_. Megalin-mediated endocytosis of 25(OH)D_3_-vitamin D binding protein complex is an important mechanism for cellular uptake of 25(OH)D_3_ in many types of cells [40,41]. 25(OH)D_3_ is more stable than 1,25(OH)_2_D_3_ as the half-life of 25(OH)D_3_ in the circulation is roughly two to three weeks while that of 1,25(OH)_2_D_3_ is only less than four hours [9,42]. Recent data suggest that 25(OH)D_3_ does not require 1,25(OH)_2_D_3_ to exert its biological action (as shown by the inhibition of 1-α hydroxylase) [13]. In addition, studies have revealed that 25(OH)D_3_ at physiological concentrations is as potent as 1,25(OH)_2_D_3_ at pharmacological concentrations in various types of cells [13,14,15,16,17,18]. Furthermore, several studies have demonstrated that 25(OH)D_3_ is an active hormone without being converted into 1,25(OH)_2_D_3_ in various types of cells [13,16,17,18,43]. 25(OH)-19-nor-D_3_ is an analog of 25(OH)D_3_ that cannot be 1α-hydroxylated. Munetsuna et al., showed that anti-proliferative activity of 25(OH)-19-nor-D_3_ is VDR dependent but 1α-hydroxylation independent [44]. In addition to its conversion into 1,25(OH)_2_D_3_, 25(OH)D_3_ and 1,25(OH)_2_D_3_ may also be converted to 24R,25(OH)_2_D and 1,24,25-(OH)_3_D_3_, respectively, by the 24-hydroxylase [43,44]. Since 24R,25(OH)_2_D and 1,24,25-(OH)_3_D_3_ have distinct biological effects documented in various tissues and cell lines [45,46], it is currently unknown to which extent 25(OH)D_3_ exerts its function directly or via its metabolites. Hence, the anabolic effects of 25(OH)D_3_ supplementation shown in this study require future in-depth analysis of metabolic pathways of metabolites, such as by using metabolomic strategies.

The status of serum concentrations of 25(OH)D_3_ is influenced by the interplay between VDBP and free 25(OH)D_3_, which may be disrupted in the setting of CKD, due to the urinary loss of VDBP [47]. The serum concentration of VDBP was elevated in CKD mice and unchanged after repletion of 25(OH)D_3_ or 1,25(OH)_2_D_3_ (Table 5). Increased serum PTH concentrations were associated with accelerated WAT browning and muscle wasting in mouse models of CKD and cancer [48,49]. Serum PTH concentrations were still significantly elevated even with successful repletion of 25(OH)D_3_ or 1,25(OH)_2_D_3_ in CKD mice (Table 5). These results show that the effects of vitamin D repletion were independent of PTH concentrations and suggest that vitamin D insufficiency, but not hyperparathyroidism, is a causal factor in CKD-associated WAT browning and muscle wasting. To exclude the effects of PTH in CKD-associated cachexia in our experiment, parathyroidectomy would have been necessary for an additional group of CKD mice, but this was beyond the scope of this study.

We demonstrated that repletion of 25(OH)D_3_ significantly attenuated or normalized cardinal features of cachexia, including food intake, weight gain, whole-body fat and lean mass content, resting metabolic rate, as well as in vivo muscle function (rotarod activity and grip strength) in CKD mice; moreover, repletion of 25(OH)D3 was significantly more effective than repletion of 1,25(OH)_2_D_3_ (Figure 1). These results may be of clinical relevance. Recent studies demonstrate the significant negative impacts of elevated resting energy expenditure in CKD patients. Elevated resting energy expenditure has been indicated in patients with end-stage renal disease and peritoneal dialysis patients [50,51]. 

Thermoregulation in adipose tissue and muscle regulates whole-body energy metabolism via the expression of UCPs [52]. Increased expression of adipose and muscle UCPs have been implicated in various disease-associated cachexia [53,54]. UCPs are mitochondrial inner membrane proteins positioned in the same membrane as the ATPase, which is also a proton channel. UCPs and ATPase work in parallel, with UCPs generating heat and ATP synthase generating ATP. Increased UCPs expression stimulates thermogenesis while inhibiting ATP synthesis [52]. UCPs content was increased while ATP was decreased in adipose tissue and muscle in CKD mice (Figure 2). Repletion of 25(OH)D_3_ normalized BAT UCP1 and gastrocnemius UCP3 content as well as significantly attenuated adipose tissue and gastrocnemius ATP content relative to repletion of 1,25(OH)_2_D_3_ in CKD mice. 25(OH)D_3_ may directly regulate the metabolism of adipose tissue. VDR and 1α−hydroxylase, the enzyme that activates 25(OH)D_3_ to 1,25(OH)_2_D_3_, were expressed in murine 3T3-L1 pre-adipocytes, BAT of mice and human adipose tissues [55,56,57]. 25(OH)D_3_ stimulated adipogenesis, presumably through the conversion to 1,25(OH)_2_D_3_. Incubation of mouse 3T3-L1 pre-adipocytes with 25(OH)D_3_ led to an accumulation of 1,25(OH)_2_D_3_ in the media [58]. Recent data also confirmed that 25(OH)D_3_ modulates UCP3 expression in muscle via the binding site consensus sequences of VDR on the UCP-3 promoter region [59].

WAT browning is a key feature for cachexia. WAT browning precedes muscle wasting in mouse models of cachexia [5,6,7,8]. Activation of Cox2/Pgf2α, as well as Tlr2, Myd88 and Traf6, promote WAT browning [24]. We showed that repletion of 25(OH)D_3_ attenuated or normalized mRNA expression of molecules (Cox2, Pgf2α, Tlr2, Myd88 and Traf6) that regulated WAT browning in inguinal WAT of CKD mice (Figure 3). In addition, we demonstrated increased expression of thermogenic genes (Ppargc1α, Pgc1α, Cidea, Prdm16, and Dio2) in inguinal WAT of CKD mice (Figure 4). We showed that repletion of 25(OH)D_3_ normalized or attenuated thermogenic gene expression in inguinal WAT of CKD mice.

Skeletal muscle has the capacity to regenerate after injury. Muscle satellite cells are precursors of skeletal muscle cells and are typically in a quiescent state but can differentiate to form muscle fibers. Satellite cells uniformly express the transcription factor pair box 7 (Pax7) and Pax7 expression is an absolute pre-requisite for skeletal muscle regeneration [60]. Pax-7 functions upstream of myogenic factors, such as Myod and Myogenin. Myod promotes the development of myogenic precursors while Myogenin stimulates the differentiation of myoblast into myocytes and myotubes [61]. Repletion of 25(OH)D_3_ significantly decreased mRNA expression of negative regulators of skeletal muscle mass (IL-1β, IL-6 and TNF-α as well as Atrogin-1, Murf-1 and Myostatin), while increasing the expression of positive regulators of skeletal muscle mass (Myod, Myogenin and Pax7) relative to repletion of 1,25(OH)_2_D_3_ in CKD mice (Figure 5).

A recent study compared the differential role of 25(OH)D_3_ and 1,25(OH)_2_D_3_ on body composition, muscle function and muscle biopsy gene expression in a cohort of healthy volunteers. These data suggest that serum concentrations of 25(OH)D_3_ have potent actions on muscle gene expression and are closely linked to body fat mass. In contrast, concentrations of serum 1,25(OH)_2_D_3_ have limited effects on gene expression associated with increased muscle strength and lean mass in women [39]. Recent data highlight the potential cytotoxicity of 1,25(OH)_2_D_3_. Srikuea et al. reported that direct intramuscular injection of supraphysiological concentration of 1,25(OH)_2_D_3_ (1 µg/kg/day for four consecutive days) decreased differentiation of satellite cells, delayed regeneration of muscle fiber and increased muscle fibrosis in a mouse model of BaCl_2_-induced muscle injury [62].

We then investigated the effects of vitamin D repletion on muscle morphology by measuring fiber size and fatty infiltration of the gastrocnemius in mice. We chose to use gastrocnemius for these studies as this muscle contains ~50% of slow twitch fibers and ~32% of fast twitch fibers in mice. Similar proportions of slow and fast twitch fibers have been reported for these hindlimb muscles in other mammals [63]. Repletion of 1,25(OH)_2_D_3_ did not improve the fiber size of the gastrocnemius in CKD mice (Figure 6). On the other hand, repletion of 25(OH)D_3_ increased the fiber size of the gastrocnemius and decreased fatty infiltration of the gastrocnemius in CKD mice (Figure 6 and Figure 7).

Muscle fibrosis has been implicated in muscle wasting [64]. We evaluated the differential effects of 25(OH)D_3_ versus 1,25(OH)_2_D_3_ repletion on the expression of muscle fibrotic genes that we have previously identified in CKD mice [12]. We demonstrated that repletion of 25(OH)D_3_ normalized muscle expression of pro-fibrotic genes (Tgfα1 and Tgif1) in CKD mice while repletion of 1,25(OH)_2_D_3_ did not (Figure 8). Furthermore, repletion of 25(OH)D_3_ significantly decreased the expression of pro-fibrotic genes (PAI-1, IL-1α, IL-1β, Agt, Ctgf, Akt1 and Smad3) while significantly increasing the expression of anti-fibrotic genes (Bmp7 and IL-13Rα2) of the gastrocnemius relative to repletion of 1,25(OH)_2_D_3_ in CKD mice. 

Finally, we evaluated the effects of vitamin D repletion on muscle transcriptome in CKD mice. Recently, we have performed RNAseq analysis in gastrocnemius muscle between 12-month-old CKD and control mice and identified the top 12 differentially expressed genes that have been associated with energy metabolism, skeletal and muscular system development and function, nervous system development and function as well as organismal injury and abnormalities [12]. In this study, we examined the effects of vitamin D repletion on gastrocnemius expression of these top 12 differentially expressed genes in younger CKD mice (three months of age at sacrifice). Repletion of 25(OH)D_3_ normalized (Atp2a2, Fhl1, Tnnc1) expression in CKD mice (Figure 9). Moreover, repletion of 25(OH)D_3_ significantly decreased expression of upregulated genes (Csrp3, Cyfip2, Myl2) while increasing the expression of downregulated genes (Atf3, Fos, Itpr1) relative to repletion of 1,25(OH)_2_D_3_ in CKD mice. Nonsignificant changes were observed in Gng2, Tpm3, and Maff muscle gene expression between repletion of 25(OH)D_3_ and 1,25(OH)_2_D_3_ in CKD mice. The functional significance of these differentially expressed muscle genes were summarized in Table 6. Decreased muscle expression of Csrp3, Fhl1, Myl2 and Tnnc1 and increased expression of Itpr1stimulate muscle regeneration and improve muscle function [26,27,30,31,32,33,34,38]. Decreased expression of Cyfip2 improves remodeling of extracellular matrix and leads to accelerated muscle regeneration [28,29]. Decreased expression of Atp2a2 and increased expression of Atf3 decreases tissue thermogenesis and improves muscle mechanical property as well as regeneration of muscle-neuron capacity [25,35]. Glucose uptake is facilitated by various tissue-specific glucose transporters. GLUT-1 transporter is the predominant isotype in rat vascular smooth muscle cells (VSMCs). Increased expression of Fos has been associated with improved glucose transport to VSMCs. Studies in cultured rat VSMCs demonstrated that increased expression of Fos preceded the increased expression of GLUT1 mRNA [37].

## 5. Conclusions

**Figure 10:**
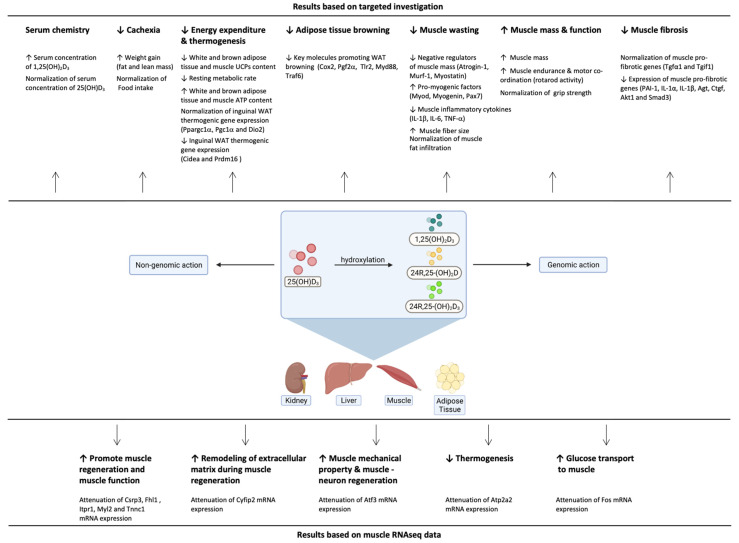
Summary of the metabolic advantages of repletion of 25(OH)D_3_ over repletion of 1,25(OH)_2_D_3_ on serum chemistry, cachexia, energy expenditure and thermogenesis, adipose tissue browning and muscle wasting in CKD mice. Created with BioRender.com, accessed 7 September 2021.

Patients with CKD have low circulating levels of 25(OH)D_3_ and 1,25(OH)_2_D_3_. The results of our investigation provide evidence that repletion of 25(OH)D_3_ exerts metabolic advantages over repletion of 1,25(OH)_2_D_3_ via multiple cellular mechanisms (Figure 10). Early detection and repletion of circulating 25(OH)D_3_ should be emphasized as an important therapeutic approach in patients with CKD based on its beneficial impact on attenuating the browning of adipose tissue and cachexia.

## Figures and Tables

**Table 1:** Serum and blood chemistry of mice. Eight-week-old CKD and sham mice were treated with 25(OH)D_3_ (25 µg/kg/day), 1,25(OH)_2_D_3_ (20 ng/kg/day) or vehicle control (ethylene glycol) for 6 weeks. Four groups of mice were included: Sham + Vehicle, CKD + Vehicle, CKD + 25(OH)D_3_ and CKD + 1,25(OH)_2_D_3_. All mice were fed *ad libitum*. Data are expressed as mean ± SEM. Results of CKD + Vehicle, CKD + 25(OH)D_3_ and CKD + 1,25(OH)_2_D_3_ mice were compared to those of Sham + Vehicle mice, respectively. ^a^ *p* < 0.05, significantly different in CKD mice than sham mice. ^b^ *p* < 0.05, significantly different in CKD + 25(OH)D_3_ or CKD + 1,25(OH)_2_D_3_ mice versus CKD + Vehicle mice. BUN, blood urea nitrogen.

	Sham + Vehicle (*n* = 6)	CKD + Vehicle (*n* = 8)	CKD + 25(OH)D_3_ (*n* = 8)	CKD + 1,25(OH)_2_D_3_ (*n* = 8)
BUN (mg/dL)	26.7 ± 3.2	54.7 ± 16.1 ^a^	64.3 ± 14.1 ^a^	62.1 ± 5.9 ^a^
Creatinine (mg/dL)	0.12 ± 0.04	0.23 ± 0.05 ^a^	0.18 ± 0.02 ^a^	0.21 ± 0.06 ^a^
Bicarbonate (mmol/L)	27.6 ± 3.2	28.1 ± 2.5	26.3 ± 2.8	27.4 ± 1.6
25(OH)D_3_ (ng/mL)	110.3 ± 28.4	43.5 ± 4.1 ^a^	69.7 ± 8.4 ^a,b^	57.6 ± 10.5 ^a^
1,25(OH)_2_D_3_ (pg/mL)	282.1 ± 56.6	124.3 ± 24.1 ^a^	146.7 ± 25.1 ^a^	176.1 ± 27.9 ^a,b^

**Table 2:** Serum and blood chemistry of mice. Eight-week-old CKD and sham mice were treated with 25(OH)D_3_ (50 µg/kg/day), 1,25(OH)_2_D_3_ (40 ng/kg/day) or vehicle control (ethylene glycol) for 6 weeks. Four groups of mice were included: Sham + Vehicle, CKD + Vehicle, CKD + 25(OH)D_3_ and CKD + 1,25(OH)_2_D_3_. All mice were fed *ad libitum*. Data are expressed as mean ± SEM. Results of CKD + Vehicle, CKD + 25(OH)D_3_ and CKD + 1,25(OH)_2_D_3_ mice were compared to those of Sham + Vehicle mice, respectively. ^a^ *p* < 0.05, significantly different in CKD mice than sham mice. ^b^ *p* < 0.05, significantly different in CKD + 25(OH)D_3_ or CKD + 1,25(OH)_2_D_3_ mice versus CKD + Vehicle mice.

	Sham + Vehicle (*n* = 6)	CKD + Vehicle (*n* = 6)	CKD + 25(OH)D_3_ (*n* = 6)	CKD + 1,25(OH)_2_D_3_ (*n* = 6)
BUN (mg/dL)	30.1± 3.8	72.3 ± 12.1 ^a^	83.1 ± 16.8 ^a^	58.6 ± 13.7 ^a^
Creatinine (mg/dL)	0.09 ± 0.02	0.28 ± 0.08 ^a^	0.27 ± 0.03 ^a^	0.26 ± 0.06 ^a^
Bicarbonate (mmol/L)	27.3 ± 1.4	27.5 ± 0.9	27.2 ± 0.8	27.8 ± 1.4
25(OH)D_3_ (ng/mL)	121.5 ± 22.1	50.7 ± 8.7 ^a^	97.6 ± 7.6 ^a,b^	60.6 ± 7.4 ^a^
1,25(OH)_2_D_3_ (pg/mL)	267.8 ± 33.6	136.4 ± 16.1 ^a^	176.8 ± 27.3 ^a^	231.1 ± 25.4 ^a,b^

**Table 3:** Serum and blood chemistry of mice. Eight-week-old CKD and sham mice were treated with 25(OH)D_3_ (75 µg/kg/day), 1,25(OH)_2_D_3_ (60 ng/kg/day) or vehicle control (ethylene glycol) for 6 weeks. Four groups of mice were included: Sham + Vehicle, CKD + Vehicle, CKD + 25(OH)D_3_ and CKD + 1,25(OH)_2_D_3_. All mice were fed *ad libitum*. Data are expressed as mean ± SEM. Results of CKD + Vehicle, CKD + 25(OH)D_3_ and CKD + 1,25(OH)_2_D_3_ mice were compared to those of Sham + Vehicle mice, respectively. ^a^ *p* < 0.05, significantly different in CKD mice than sham mice. ^b^ *p* < 0.05, significantly different in CKD + 25(OH)D_3_ or CKD + 1,25(OH)_2_D_3_ mice versus CKD + Vehicle mice.

	Sham + Vehicle (*n* = 4)	CKD + Vehicle (*n* = 6)	CKD + 25(OH)D_3_ (*n* = 8)	CKD + 1,25(OH)_2_D_3_ (*n* = 8)
BUN (mg/dL)	27.5 ± 6.4	65.8 ± 11.4 ^a^	67.8 ± 5.1 ^a^	75.4 ± 9.3 ^a^
Creatinine (mg/dL)	0.11 ± 0.02	0.18 ± 0.02 ^a^	0.21 ± 0.03 ^a^	0.23 ± 0.07 ^a^
Bicarbonate (mmol/L)	27.6 ± 2.2	27.8 ± 2.3	27.3 ± 2.1	27.3 ± 1.6
25(OH)D_3_ (ng/mL)	113.5 ± 11.9	50.7 ± 9.4 ^a^	115.3 ± 7.6 ^b^	67.4 ± 11.5 ^a^
1,25(OH)_2_D_3_ (pg/mL)	267.3 ± 23.1	116.4 ± 17.2 ^a^	213.4 ± 21.3 ^a,b^	257.1 ± 25.2 ^b^

**Table 4:** Serum and blood chemistry of mice. Eight-week-old CKD and sham mice were treated with 25(OH)D_3_ (75 µg/kg/day), 1,25(OH)_2_D_3_ (60 ng/kg/day) or vehicle control (ethylene glycol) for 6 weeks. Six groups of mice were included: Sham + Vehicle, Sham + 25(OH)D_3_, Sham + 1,25(OH)_2_D_3_, CKD + Vehicle, CKD + 25(OH)D_3_ and CKD + 1,25(OH)_2_D_3_. All mice were fed *ad libitum*. Data are expressed as mean ± SEM. Results of CKD + Vehicle, CKD + 25(OH)D_3_ and CKD + 1,25(OH)_2_D_3_ mice were compared to those of Sham + Vehicle, Sham + 25(OH)D_3_ and Sham + 1,25(OH)_2_D_3_ mice, respectively. ^a^ *p* < 0.05, significantly higher in CKD mice than sham mice. ^b^ *p* < 0.05, significantly lower in CKD mice than sham mice. Results of CKD + Vehicle were compared to those of CKD + 25(OH)D_3_ or CKD + 1,25(OH)_2_D_3_ mice. ^c^ *p* < 0.05, significantly different in CKD + 25(OH)D_3_ mice or CKD + 1,25(OH)_2_D_3_ mice than CKD + Vehicle mice. Moreover, results of CKD + 25(OH)D_3_ mice were compared to those of CKD + 1,25(OH)_2_D_3_ mice. ^d^ *p* < 0.05, significantly different between CKD + 25(OH)D_3_ mice and CKD + 1,25(OH)_2_D_3_ mice.

	Sham + Vehicle (*n* = 14)	Sham + 25(OH)D_3_ (*n* = 13)	Sham + 1,25(OH)_2_D_3_ (*n* = 11)	CKD + Vehicle (*n* = 13)	CKD + 25(OH)D_3_ (*n* = 14)	CKD + 1,25(OH)_2_D_3_ (*n* = 13)
BUN (mg/dL)	26.5 ± 5.6	32.4 ± 3.1	27.3 ± 4.3	57.5 ± 6.5 ^a^	68.7 ± 6.9 ^a^	58.7 ± 6.4 ^a^
Creatinine (mg/dL)	0.09 ± 0.03	0.12 ± 0.04	0.09 ± 0.02	0.21 ± 0.04 ^a^	0.17 ± 0.04 ^a^	0.19 ± 0.05 ^a^
Bicarbonate (mmol/L)	27.5 ± 1.2	27.8 ± 1.4	27.9 ± 1.2	26.8 ± 1.5	26.5 ± 1.7	27.9 ± 2.3
25(OH)D_3_ (ng/mL)	109.5 ± 14.4	112.4 ± 16.5	124.3 ± 15.6	54.7 ± 11.6 ^b^	105.4 ± 13.8 ^c,d^	53.8 ± 9.8 ^b^
1,25(OH)_2_D_3_ (pg/mL)	256.7 ± 21.3	243.5 ± 24.3	265.4 ± 19.5	136.5 ± 15.3 ^b^	185.7 ± 15.5 ^b,c^	246.9 ± 21.4 ^c,d^

**Table 5:** Serum and blood chemistry of mice. Eight-week-old CKD and sham mice were treated with 25(OH)D_3_ (75 µg/kg/day), 1,25(OH)_2_D_3_ (60 ng/kg/day) or vehicle control (ethylene glycol) for 6 weeks. Six groups of mice were included: Sham + Vehicle, Sham + 25(OH)D_3_, Sham + 1,25(OH)_2_D_3_, CKD + Vehicle, CKD + 25(OH)D_3_ and CKD + 1,25(OH)_2_D_3_. CKD + Vehicle mice were fed *ad libitum* whereas all other groups of mice were given the equivalent amount of energy intake as those of CKD + Vehicle mice. Data are expressed as mean ± SEM. Results of CKD + Vehicle, CKD + 25(OH)D_3_ and CKD + 1,25(OH)_2_D_3_ mice were compared to those of Sham + Vehicle, Sham + 25(OH)D_3_ and Sham + 1,25(OH)_2_D_3_ mice, respectively. ^a^ *p* < 0.05, significantly higher in CKD mice than sham mice. ^b^ *p* < 0.05, significantly lower in CKD mice than sham mice. Results of CKD + Vehicle were compared to those of CKD + 25(OH)D_3_ or CKD + 1,25(OH)_2_D_3_ mice. ^c^ *p* < 0.05, significantly different in CKD + 25(OH)D_3_ mice or CKD + 1,25(OH)_2_D_3_ mice than CKD + Vehicle mice. Results of CKD + 25(OH)D_3_ mice were compared to those of CKD + 1,25(OH)_2_D_3_ mice. ^d^ *p* < 0.05, significantly different between CKD + 25(OH)D_3_ mice and CKD + 1,25(OH)_2_D_3_ mice. BUN, blood urine nitrogen; Ca, calcium; Pi, inorganic phosphate; PTH, parathyroid hormone; VDBP, vitamin D binding protein.

	Sham + Vehicle (*n* = 11)	Sham + 25(OH)D_3_ (*n* = 11)	Sham + 1,25(OH)_2_D_3_ (*n* = 11)	CKD + Vehicle (*n* = 14)	CKD + 25(OH)D_3_ (*n* = 12)	CKD + 1,25(OH)_2_D_3_ (*n* = 13)
BUN (mg/dL)	27.4 ± 4.6	29.5 ± 3.7	28.5 ± 5.6	64.6 ± 6.6 ^a^	72.5 ± 8.7 ^a^	68.7 ± 9.9 ^a^
Ca (mg/dL)	11.2 ± 0.4	11.9± 0.5	11.4 ± 0.7	9.7 ± 0.4 ^b^	8.9 ± 0.7 ^b^	9.4 ± 0.5 ^b^
Creatinine (mg/dL)	0.06 ± 0.04	0.09 ± 0.03	0.07 ± 0.02	0.17± 0.05 ^a^	0.18 ± 0.07 ^a^	0.21 ± 0.07 ^a^
Bicarbonate (mmol/L)	28.5 ± 2.3	28.3 ± 2.2	28.3 ± 2.1	27.8 ± 2.5	26.8 ± 2.4	26.7 ± 2.1
25(OH)D_3_ (ng/mL)	115.5 ± 18.7	107.8 ± 12.6	123.2 ± 21.5	47.8 ± 9.8 ^b^	124.6 ± 11.5 ^c,d^	50.1 ± 11.4 ^b^
1,25(OH)_2_D_3_ (pg/mL)	268.7 ± 17.4	277.4 ± 31.7	247.8 ± 23.6	109.6 ± 21.5 ^b^	207.8 ± 21.5 ^c^	276.5 ± 33.7 ^c,d^
Pi (mg/dL)	7.5 ± 0.3	7.3 ± 0.6	7.4 ± 0.6	9.7 ± 0.5 ^a^	9.8 ± 0.6 ^a^	9.6 ± 0.4 ^a^
PTH (pg/mL)	118.3 ± 15.7	105.7 ± 17.8	109.5 ± 19.8	326.7 ± 23.6 ^a^	283 ± 32.5 ^a^	275.5 ± 27.3 ^a^
VDBP (ug/mL)	398.5 ± 27.5	408.4 ± 26.7	387.5 ± 27.6	591.5 ± 25.6 ^a^	581.8 ± 23.1 ^a^	625.8 ± 25.7 ^a^

**Table 6:** Repletion of 25-hydroxyvitamin D_3_ normalizes or attenuated expression of important muscle genes in CKD mice. Functional significance for each of these differentially expressed muscle genes is listed. DEG, differentially expressed genes.

Upregulated DEG	Functional Significance and References
Atp2a2	associated with UCP-1 independent beige thermogenesis [25]
Csrp3	associated with skeletal muscle dystrophy [26,27]
Cyfip2	associated with muscle atrophy [28,29]
Fhl1	activates myostatin signaling and promotes atrophy in skeletal muscle [30]
Myl2	associated with muscle cycling kinetics [31,32,33]
Tnnc1	biomarker for muscle depolarization [34]
**Downregulated DEG**	**Functional Significance and References**
Atf3	Biomarker for neural injury, associated with reduced regeneration of neurons [35]
Fos	associated with decreased skeletal muscle regeneration [36]
Itpr1	impairs glucose transport in muscle [37] associated with decreased muscle regeneration and mitochondrial dysfunction in myopathy patients [38]

## Data Availability

The authors confirm that the data supporting the findings of this study are available within the article and its Supplementary Materials. Additional raw data supporting the findings of this study are available from the corresponding author (R.H.M) on request.

## Supplementary Materials

The following are available online at https://www.mdpi.com/article/10.3390/cells10123382/s1, Table S1: Immunoassay information for blood and serum chemistry, muscle adenosine triphosphate content as well as muscle and adipose tissue protein analysis; Table S2: PCR primer information.
