# Supplementary material for: Differential Effects of 25-Hydroxyvitamin D3 versus 1α 25-Dihydroxyvitamin D3 on Adipose Tissue Browning in CKD-Associated Cachexia
Source: Cells. 2021 Dec 1;10(12):3382. doi: 10.3390/cells10123382 (PMC8699879; doi:10.3390/cells10123382)
Supplement: Supplementary file 1 [file cells-10-03382-s001.zip › cells-1426632-supplementary.pdf]

Article

Differential Effects of 25-hydroxyvitamin D_3 Versus 1α, 25-dihydroxyvitamin D_3 on Adipose Tissue Browning in CKD-Associated Cachexia

Robert H. Mak [1*], Uwe Querfeld [2], Alex Gonzalez [1], Sujana Gunta[1,3], Wai W. Cheung [1]

[1] Division of Pediatric Nephrology, Rady Children's Hospital, University of California, San Diego
92093-0831, USA; romak@health.ucsd.edu (R.H.K.); w5cheung@health.ucsd.edu (W.W.C.); alg022@health.ucsd.edu (A.G.)
[2] Department of Paediatric Nephrology, Charité Children's Hospital, Augustenburger Platz 1, 13353 Berlin, Germany; uwe.querfeld@charite.de (U.Q.)
[3] Pediatric Services, Vista Community Clinic, Vista, CA 92084; sujana.kaushik@gmail.com (S.G.)
* Correspondence: romak@health.ucsd.edu; Tel.: +1-858-822-6717; Fax: +1-858-822-6776

Supplemental information

Supplemental Table S1: Immunoassay information for blood and serum chemistry, muscle adenosine triphosphate content as well as muscle and adipose tissue protein analysis.

Blood & Serum chemistry	Assay information
Bicarbonate, BUN, Ca & Pi	VetScan Comprehensive Diagnostic Profile, Abaxix, 500-0038
Creatinine	LC-MS/MS method (reference # 20)
25(OH)D_3	IDS, AC-35F1
1,25$(OH)_2D_3$	IDS, AC-62F1
PTH	Immutopics, 60-2305
VDBP	R&D Systems, MVDBPO

Muscle & adipose tissue	Assay information
ATP Assay Kit (Colorimetric / Fluorometric)	Abcam, ab83355
Mouse UCP1 ELISA kits	Aviva Systems Biology, OKCD02970
Mouse UCP3 ELISA kits	Aviva Systems Biology, OKEH05259

Supplemental Table S2: PCR primer information

Gene	Forward primer sequence	Reverse primer sequence
Agt	TCTCCTTTACCACAACAAGAGCA	CTTCTCATTCACAGGGGAGGT
Akt1	CTGCTCCTAGTCCACCACCT	AGAGACCTCCATTATCGCTACC
Atf3	GAGGATTTTGCTAACCTGACACC	TTGACGGTAACTGACTCCAGC
Atp2α2	GAGAACGCTCACACAAAGACC	CAATTCGTTGGAGCCCCAT
Atrogin-1	CAGCTTCGTGAGCGACCTC	GGCAGTCGAGAAGTCCAGTC
Bmp7	ACGGACAGGGCTTCTCCTAC	ATGGTGGTATCGAGGGTGGAA
CD137	CGTGCAGAACTCCTGTGATAAC	GTCCACCTATGCTGGAGAAGG
Cidea	TGACATTCATGGGATTGCAGAC	GGCCAGTTGTGATGACTAAGAC
Cyfip2	ATGACCACCCACGTCACTTTG	CCTGTCCTCGAAGTTCGTGTC
Cox2	AACCCAGGGGATCGAGTGT	CGCAGCTCAGTGTTTGGGAT
Csrp3	GGGGGAGGTGCAAAATGTG	CAGGCCATGCAGTGGAAACA
Ctgf	GTGGAATATTGCCGGTGCA	CCATTGAAGCATCTTGGTTCG
Dio2	AATTATGCCTCGGAGAAGACCG	GGCAGTTGCCTAGTGAAAGGT
Fhl1	GACTGCCGCAAGCCCATAA	CCAAGGGGTGAAGGCACTT
Fos	TTGAGCGATCATCCCGGTC	GCGTGAGTCCATACTGGCAAG
Gng2	ACCGCCAGCATAGCACAAG	AGTAGGCCATCAAGTCAGCAG
IL-1α	ACGGCTGAGTTTCAGTGAGACC	CACTCTGGTAGGTGTAAGGTGC
IL-1β	GCAACTGTTCCTGAACTCAACT	ATCTTTTGGGGTCCGTCAACT
IL-6	TAGTCCTTCCTACCCCAATTTCC	TTGGTCCTTAGCCACTCCTTC
IL-13Rα2	ACCGAAATGTTGATAGCGACAG	ACAATGCTCTGACAAATGCGTA
Itpr1	CGTTTTGAGTTTGAAGGCGTTT	CATCTTGCGCCAATTCCCG
Maff	AGGAGGAGGTCATCCGACTG	CTTCTCGCTCTCCAGAATGTG
Murf-1	GTGTGAGGTGCCTACTTGCTC	GCTCAGTCTTCTGTCCTTGGA
Myd88	TCATGTTCTCCATACCCTTGGT	AAACTGCGAGTGGGGTCAG
Myl2	ATCGACAAGAATGACCTAAGGGA	ATTTTTCACGTTCACTCGTCCT
Myod	CCACTCCGGGACATAGACTTG	AAAAGCGCAGGTCTGGTGAG
Myogenin	GAGACATCCCCCTATTTCTACCA	GCTCAGTCCGCTCATAGCC
Myostatin	AGTGGATCTAAATGAGGGCAGT	GTTTCCAGGCGCAGCTTAC
PAI-1	TCTGGGAAAGGGTTCACTTTACC	GACACGCCATAGGGAGAGAAG
Pax7	TCTCCAAGATTCTGTGCCGAT	CGGGGTTCTCTCTCTTATACTCC
Pgc1α	TATGGAGTGACATAGAGTGTGCT	CAGGAGTTGATTCCAGACAGGTA
Pgf2α	CTGGACTCATCGCAAACACAA	AGGAAGCCTTTGACTTCTGTCTA
Ppargc1α	AGAGCCCCATCTGTCCTCTC	ACTGGTAGTCTGCAAAACCAAA
Prdm16	CCCCACATTCCGCTGTGAT	CTCGCAATCCTTGCACTCA
Tbx-1	CTGTGGGACGAGTTCAATCAG	TTGTCATCTACGGGCACAAAG
Tgfα1	TGATACGCCTGAGTGGCTGTCT	CACAAGAGCAGTGAGCGCTGAA
Tgif1	GAACACAGATACAACGCCTATCC	CCATCCTTTCTCAGCATGTCAG
Timp3	CTTCTGCAACTCCGACATCGT	GGGGCATCTTACTGAAGCCTC
Tlr2	GCAAACGCTGTTCTGCTCAG	AGGCGTCTCCCTCTATTGTATT
Tmem26	TTCCTGTTGCATTCCCTGGTC	GCCGGAGAAAGCCATTTGT
Tnf-α	CCCTCACACTCAGATCATCTTCT	GCTACGACGTGGGCTACAG
Tnnc1	GCGGTAGAACAGTTGACAGAG	CCAGCTCCTTGGTGCTGAT
Tpm3	ACCACCATCGAGGCGGTAA	CCCTTTCCTCCGCATCATCA
Traf6	AAAGCGAGAGATTCTTTCCCTG	ACTGGGGACAATTCACTAGAGC
Smad3	AAAGCGAGAGATTCTTTCCCTG	ACTGGGGACAATTCACTAGAGC
Gapdh (internal control)	AGGTCGGTGTGAACGGATTTG	TGTAGACCATGTAGTTGAGGTCA
